# Neuroimmune Evasion of Zika Virus to Facilitate Viral Pathogenesis

**DOI:** 10.3389/fcimb.2021.662447

**Published:** 2021-10-26

**Authors:** Xiaochun Xie, Jianxiong Zeng

**Affiliations:** ^1^ Key Laboratory of Animal Models and Human Disease Mechanisms of the Chinese Academy of Sciences, Kunming Institute of Zoology-The Chinese University of Hong Kong (KIZ-CUHK) Joint Laboratory of Bioresources and Molecular Research in Common Diseases, Kunming Institute of Zoology, Chinese Academy of Sciences, Kunming, China; ^2^ Kunming National High-level Biosafety Research Center for Non-Human Primates, Center for Biosafety Mega-Science, Kunming Institute of Zoology, Chinese Academy of Sciences, Kunming, China; ^3^ National Resource Center for Non-Human Primates, National Research Facility for Phenotypic and Genetic Analysis of Model Animals (Primate Facility), Kunming Institute of Zoology, Chinese Academy of Sciences, Kunming, China

**Keywords:** ZIKV, capsid, NS3, NS4A/4B, pathogenesis, NS5, immune evasion

## Abstract

Zika virus (ZIKV), which preferentially targets neural stem and progenitor cells (NSCs) especially in developing brain, is causally associated with fetal microcephaly, intrauterine retardation, and other congenital malformations in humans. However, there are, so far, no effective drugs and vaccines against ZIKV epidemics, warranting an enhanced understanding of ZIKV biology. Immune response is essential for neuronal cells to combat viral invasion. In turn, neurotropic ZIKV has developed a complex strategy of neuroimmune evasion to facilitate viral pathogenesis, especially developmental impairment in embryonic brain. Here, we review not only overall knowledge of ZIKV-related immune responses, but also current advances in our understanding of immune evasion in ZIKV infection. We also review several specific mechanisms underlying ZIKV protein-mediated immune evasion for viral pathogenesis.

## Introduction

Zika virus (ZIKV) is a single-stranded positive-sense RNA virus that belongs to the genus *Flavivirus* of the family *Flaviviridae*. The RNA genome is translated directly into one large polyprotein, which produces 10 viral proteins (3 structural and 7 nonstructural proteins) *via* proteolytic cleavage by host and viral enzymes. The *Flavivirus* genus includes more than 50 arthropod-borne viruses with public health importance including Dengue virus (DENV), West Nile virus (WNV), Yellow fever virus (YFV), and Japanese encephalitis (JEV). ZIKV was originally isolated from a sentinel monkey in the Zika forest or Uganda in 1947 (Dick et al., 1952). ZIKV infection in human was just sporadically reported in a few African and Asian countries with mild symptoms (Weaver et al., 2016) and associated with Guillain–Barre disease (Cao-Lormeau et al., 2016). However, ZIKV unexpectedly emerged since 2015 and suddenly became a global public health threat due to its explosive outbreaks in the Americas. In a short time, ZIKV was considered as the etiological agent for fetal microcephaly and congenital Zika syndrome (CZS) (Rasmussen et al., 2016; Hoen et al., 2018). In the following years, ZIKV spread to 86 countries or territories worldwide, and it was estimated that ~3.6 billion people are living in areas at risk for transmission (Baud et al., 2017).

ZIKV is able to lead to devastating fetal microcephaly in infants born from infected pregnant mothers (Musso and Schot, 2016). Thousands of infants born from ZIKV-infected mothers in the Americas had impaired neurodevelopment with thinner cortical layers (Orioli et al., 2017). ZIKV is characterized by the intrinsic tropism for neural stem and progenitor cells (NSCs) in cell cultures, brain organoids, and fetal brain slices (Cugola et al., 2016; Dang et al., 2016; Garcez et al., 2016; Liang et al., 2016; Qian et al., 2016; Tang et al., 2016). ZIKV infection results in the impairment of NSC proliferation and differentiation, induces cell death, and ultimately causes cerebral developmental deficits (Shao et al., 2016; Nielsen-Saines et al., 2019; Zeng et al., 2020).

Immune response is critical for antagonizing neurotropism virus infection. The host utilizes multiple pattern recognition receptors (PRRs) to patrol diverse pathogen-associated molecular patterns (PAMPs), consequently activating antiviral responses including the production of interferons (IFNs) (Meylan et al., 2006). For example, retinoic acid-inducible gene-I (RIG-I) senses ZIKV-RNA and triggers MAVS-TBK1-IRF3 signaling and type I IFN production (Chazal et al., 2018). In order to evade the IFN-mediated surveillance, ZIKV evolved specific non-structural (NS) proteins for viral evasion, including NS1 (Xia et al., 2018; Zheng et al., 2018), NS3 (Riedl et al., 2019), and NS5 (Grant et al., 2016; Kumar et al., 2016). Notably, mammalian multipotent stem cells including NSCs produce little IFNs and respond poorly to IFN treatment compared to somatic cells (Hong and Carmichael, 2013; Wu et al., 2019). Instead, these cells rely on other antiviral machineries such as RNA interference (RNAi) (Ding and Voinnet, 2007). Therefore, this short review seeks to highlight recent advances in our understanding of ZIKV protein-mediated neuroimmune evasion, which contributes to virus pathogenicity.

## ZIKV Capsid Targets Dicer to Inhibit miRNA Biogenesis in Developing Brain

In many eukaryotes, RNAi is a critical cellular mechanism by which short RNA oligos specifically pair with targeted mRNAs to regulate or inhibit their translation or gene expression, thereby maintaining homeostatic function in cells. Dicer as a critical microRNA (miRNA) biogenesis enzyme, is indispensable for the RNAi pathway, where it functions to cleave double-strand RNAs (dsRNAs) or stem-loop structure of pre-miRNAs into short dsRNAs, which are then loaded on the RNA-induced silencing complex (RISC) and processed into ~22-nucleotide (nt) mature miRNAs (Jinek and Doudna, 2009). Consequently, the mature miRNA pairs with the targeted complementary sequence in the 3’ untranslated region (UTR) of an mRNA molecule and causes a cleavage by Argonaute 2 (Ago2), the catalytic factor in the RISC, consequently resulting in the post-transcriptional gene silencing. Disruption of cellular miRNA homeostasis has been shown to be closely related to numerous diseases such as neurodevelopment deficits while miRNAs participate in almost every cellular process. Although type I IFN response provides a main protection against microbial invasion in most somatic cells, anti-viral RNAi especially miRNAs remains dominantly active in mammalian multipotent stem cells (Li et al., 2013; Maillard et al., 2013) since these stem cells have intrinsically less ability to respond to IFN treatment than other somatic cell types (Hong and Carmichael, 2013; Wu et al., 2018). ZIKV infection has been reported to disrupt the host miRNA profile in mammalian cells, and importantly neuronal *Dicer* deficient mice exhibit neurodevelopment disorders including microcephaly (Davis et al., 2008), strongly suggesting the potential correlation between Dicer dysfunction and ZIKV pathogenesis.

In a screening of the required host factors of ZIKV protein partners in NSCs to uncover the roles of the viral proteins in the context of ZIKV infection, ribonuclease III-like enzyme Dicer is the top hit within the resulted ZIKV–host interactome (Zeng et al., 2020). While Dicer is proven to be necessary for ZIKV replication by utilizing Dicer knock-outed mouse NSCs, it is intriguing that viral capsid-mediated interaction with Dicer exists only in ZIKV, but not in other flaviviruses including DENV2, WNV, JEV, YFV, JEV, HCV, OHFV, and TBEV. Such specific interaction also expectedly leads to the phenotype that only ZIKV capsid is able to suppress Dicer enzymatic activity by utilizing common Dicer substrates like shRNA, pre-miRNA, or double-strand RNA. In alignment comparison analysis of viral capsid between ZIKV, DENV2, WNV, and JEV, one capsid mutant (H41R) was successfully selected and completely abolished capsid–Dicer interaction, consequently losing the ability to inhibit Dicer enzymatic activity. Dicer is responsible for miRNA biogenesis in mammalian cells (Zeng et al., 2020). As a result, wild-type ZIKV (ZIKV-WT) infection led to significant reduction of total miRNA reads, and, however, such phenotype was absent in rescued ZIKV-H41R mutant virus-infected NSCs. Importantly, ZIKV-WT infection resulted in the downregulation of a panel of miRNAs including let-7a, miR-9, miR-17, and miR-19a, which has been shown to be important for neurogenesis and neurodevelopment. Similar miRNA biogenesis phenotype was also observed in an animal model of *in utero* ZIKV injection (Zeng et al., 2020). As expected, utilization of this mouse model also helps confirm that ZIKV capsid protein enabled to induce neurogenic deficits and corticogenesis impairment by directly targeting Dicer in developing brains. It has been known that the dysfunction to regulate miRNA homeostasis is closely linked to a large group of human diseases (Mendell and Olson, 2012). One example is that Dicer knockdown is associated with microcephaly-like phenotypes in an animal model (Davis et al., 2008). It has been proven that fetal brain development required the critical individual miRNAs like let-7a, miR-9, miR-17, and miR-19a (Rajman and Schratt, 2017). Accordingly, ZIKV infection-induced impairment of cellular miRNA homeostasis has been reported in human NSCs (Dang et al., 2019), astrocyte-like SVG-A cells (Kozak et al., 2017), and mosquitoes (Saldana et al., 2017). On the one hand, ZIKV-capsid specific association with Dicer represents a unique mechanism on why only ZIKV in flavivirus is related to microcephaly in clinic. On the other hand, miRNAs not only have antiviral function especially in neural stem cells but also are essential for normal neurogenesis, and thus ZIKV capsid achieves these two tasks by specifically targeting Dicer (Zeng et al., 2020).

**Figure 1:**
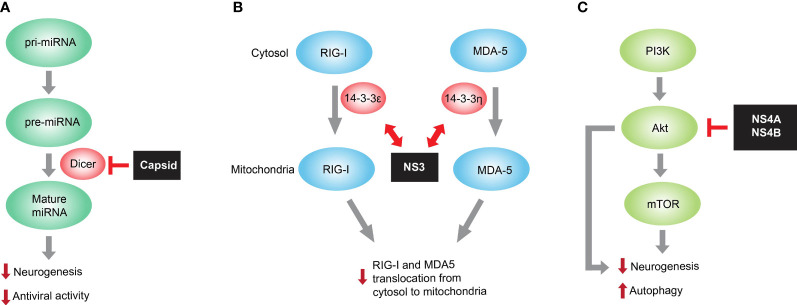
Representative ZIKV protein mediated immune evasion for facilitating viral pathogenesis. **(A)** ZIKV capsid interacts with Dicer, the host endoribonuclease responsible for producing mature miRNA from pre-miRNA, and dampens global miRNA production in neural stem cells. Because miRNA is necessary for both neurogenesis and antiviral function, the capsid-mediated miRNA inhibition causes neurogenesis impairment and reduced miRNA-mediated antiviral activity in developing brain. **(B)** ZIKV NS3 encodes a highly conserved 14-3-3-binding motif, which enables it to interact with 14-3-3ϵ/η required for translocation of RNA sensor RIG-I and MDA5, respectively, from cytosol to mitochondria. Such interaction prevents the translocation and thus RIG-I- and MDA-mediated antiviral interferon responses. **(C)** mTOR is a host factor necessary for neurogenesis and autophagy inhibition. ZIKV NS4A and NS4B inhibit Akt-mTOR signaling with unknown mechanisms and consequently impair neurogenesis and promote autophagy, thereby contributing to ZIKV pathogenesis in developing brain.

ZIKV capsid hijacks host Dicer and suppresses its enzymatic activity to facilitate immune evasion and consequently disrupt cortical development (**Figure 1A**). As a viral structural protein, capsid is packaged into mature virion particle during virus assembly and is released right after virus entry. As a result, the capsid protein may not only wrap ZIKV RNA genome and protect it from outside the cells, but also initiate immune evasion by targeting Dicer-related RNAi pathway preceding the translation of viral genes. Therefore, ZIKV capsid targeting Dicer represents an elegant mechanism for ZIKV immune evasion in developing brain.

## ZIKV Infection Induces Anti-Viral Small Interfering RNAs in Pluripotent Cells

RNAi is an evolutionarily conserved post-transcriptional gene silencing mechanism. Besides host microRNAs (miRNAs) regulating mRNA homeostasis and fine-tuning gene expression, virus-derived small interfering RNAs (vsiRNAs) can also be produced in infected host cells (Guo et al., 2019). In fact, vsiRNA production is an ancient innate immune response, defending the plants and animals from virus infection (Maillard et al., 2013; tenOever, 2016). In mammals, the physiological importance of vsiRNAs in somatic cells perhaps is still under investigation. It has been proposed that canonical vsiRNAs are generated by Dicer from double-stranded viral replicative intermediates, as evidenced by complementary pairs of vsiRNAs mapped successively to the viral RNA genome (Maillard et al., 2013). Once produced, vsiRNAs are loaded onto AGO family proteins of the RISCs to initiate the cleavage of host target genes and cognate viral RNAs (Guo et al., 2019). ZIKV infection has been demonstrated to induce abundant vsiRNAs in NSCs and hNPCs (Xu et al., 2019; Zeng et al., 2021). Zeng et al. reported not only that vsiRNA is a limiting factor for ZIKV infection in NSCS, but also that the total 29 vsiRNAs across the ZIKV genome through AGO-associated RNA-seq. More importantly, the production of these vsiRNAs is demonstrated to be dependent on Dicer. The vsiRNA-p18, the most abundant one within the identified 29 vsiRNAs, was detectable in ZIKV-infected NSCs while its physiologically antiviral effect remains unknown (Zeng et al., 2021). Similar evidence was also shown by Xu et al. that ZIKV infection induced abundant vsiRNA productions in human neural progenitors by direct deep RNA sequencing on ZIKV-infected cells. Similarly, the vsiRNAs’ physiological importance was uncovered by the fact that the ablation of key RNAi machinery components greatly facilitates ZIKV replication, and that increased anti-ZIKV activity in hNPCs was observed in the treatment of the enoxacin, a known RNAi enhancer (Xu et al., 2019). Although the advances described above have been achieved, further studies are needed to investigate how these vsiRNAs are produced and what are the physiological functions of these vsiRNAs.

## ZIKV NS3 Antagonizes RIG-I-/MDA5-Mediated Innate Immunity

Innate immune sensors along with other critical transcription factors involved in interferon (IFN) signaling are necessary to restrict ZIKV pathogenesis (Serman and Gack, 2019). Upon viral RNA sensing, RIG-I and MDA5, both of the RIG-I-like receptor (RLR) family, translocate from the cytosol to the mitochondria and chemically activate kinase transcription factors including TBK1, IRF3, and IRF7. These transcription factors enter the nucleus to initiate IFN response, upregulate a large panel of IFN-stimulated genes (ISGs), and finally trigger to form antiviral status (Bowen et al., 2017; Chazal et al., 2018; Hertzog et al., 2018). Thereof, 14-3-3 family contains several immune-related protein members and translocates the RLR sensors to the targeted organelles in innate immunity. For example, 14-3-3ϵ facilitates the translocation from cytosol to mitochondria of the RIG-I, and 14-3-3η promotes the translocation of MDA5 to mitochondria and consequently enhances antiviral IFN response (Liu et al., 2012; Lin et al., 2019). ZIKV has been developed to inhibit or postpone IFN production and IFN-induced signaling such as ISG expression. One delicate study (Riedl et al., 2019) has shown that the ZIKV NS3 protein antagonized RIG-I- and MDA5-mediated anti-viral cascades *via* disguising itself to competitively associate with the 14-3-3 (**Figure 1B**). Interestingly, a NS3 mutation that fails to bind 14-3-3ϵ/η was defined and, as a result, the rescued NS3 mutation-containing recombinant ZIKV triggered enhanced anti-viral IFN response and consequently displayed the reduced replication capacity in SVGA cells. Interestingly, the viral protein-mediated mechanism targeting 14-3-3 is also present in other flaviviruses including DENV and WNV (Liu et al., 2012), and further delicate investigation of this immune evasion mechanism helps to look for molecule-targeting therapies or develop novel attenuated virus as a targeting strain for new vaccines.

## ZIKV NS4A/4B Deregulates Akt-mTOR Signaling to Inhibit Neurogenesis

The PI3K-Akt-mTOR pathway is one of the cellular signaling pathways indispensable for neurogenesis and migration (Lee, 2015). Prenatal development is the most active stage regarding neurogenesis that populates the developing brain with neurons (Gotz and Huttner, 2005). Dysfunction in neurogenesis and differentiation could cause neurodevelopmental disorders such as microcephaly in humans (Ming and Song, 2011). Specifically, genetic mutations in the PI3K-Akt-mTOR pathway may be present in brain overdevelopment syndromes including megalencephaly-capillary malformation (MCAP), and megalencephaly-polydactyly-polymicrogyria-hydrocephalus (MPPH) (Mirzaa et al., 2013). Instead, mechanistic target of rapamycin (mTOR)-targeted suppression in developing brain caused microcephaly (Cloetta et al., 2013). Akt is an upstream molecule of mTOR and is the central player of the PI3K pathway. Interestingly, non-functional Akt mutation also caused microcephaly in humans, and instead, activating Akt mutation led to megalencephaly (Nellist et al., 2015). Viral pathogens have been reported to target the PI3K-Akt-mTOR pathway for their multiplication and pathogenicity in mammals (Buchkovich et al., 2008).

ZIKV, DENV, and HCV have been reported to hijack host cellular autophagy for viral multiplication (Heaton and Randall, 2010; Hamel et al., 2015). The mTOR kinase is the central player for autophagy induction, with activation of mTOR by Akt and MAPK inhibiting autophagy. Instead, AMPK and p53 signaling induced mTOR inactivation and facilitates autophagy (Jung et al., 2010). mTOR inactivation triggers downstream serine/threonine kinase UNC-51-like kinase-1 (ULK1), the mammalian homolog of yeast Atg1, which facilitates class III PI3K complex formation and finally induces autophagosome formation (Kim et al., 2011). Consistently, autophagy provides host protection against viral infection. Herpesviruses such as Kaposi’s sarcoma-associated herpesvirus (KSHV) and Epstein–Barr virus (EBV) inhibit cellular autophagy, utilizing viral proteins for their persistent infection establishment (Lee et al., 2009; Williams and Taylor, 2012; Liang et al., 2013).

Human neural stem cells and iPSC-derived neural organoids are known to be vulnerable to ZIKV infection (Garcez et al., 2016; Qian et al., 2016; Tang et al., 2016). This suggests the causal link between ZIKV infection and human microcephaly through neurogenesis suppression. *Via* two primary isolates of fetal neural stem cells (fNSCs), the study showed that the mechanism underlying ZIKV infection might destroy fetal brain development (Liang et al., 2016). Specifically, impairment of neurosphere growth and neural differentiation as well as aberrant autophagy were induced by ZIKV infection of human fNSCs. In the screening of individual ZIKV protein, it is interesting that both NS4A and NS4B synergistically inhibited host Akt-mTOR signaling, impaired the neurogenesis of human fNSCs, and upregulated autophagy (**Figure 1C**). The resulting promotion of viral replication in turn caused more impairment of neural development. Thus, ZIKV NS4A and NS4B are potential virulence determinants of viral neuroimmune evasion and viral pathogenesis, discovering the promising targets for anti-ZIKV therapeutic interventions.

## ZIKV Inhibits the cGAS-STING Pathway

cGAS-STING as a critical innate immunity pathway has been shown to antagonize flavivirus infection (Schoggins et al., 2015; Aguirre et al., 2017; Sun et al., 2017) and accordingly flaviviruses also evolved multiple strategies to evade this pathway. cGAS-generated cGAMP-mediated IFN signaling in response to ZIKV infection in human fibroblasts was reduced while this reduction could not be observed in mouse fibroblasts. Mechanistically, ZIKV NS2b3 protein utilizes its enzymatic activity to cleave human but not mouse STING (Ding et al., 2018). However, although fibroblasts from rhesus macaque and chimpanzee are permissive to ZIKV infection, STING protein from these species can be cleaved by ZIKV NS2b3, suggesting the existence of another unknown mechanism. An interesting study reported that ZIKV NS1 not only triggered NLRP3 inflammasome activation but also contributed to caspase-1 stabilization, which facilitated caspase-1-mediated cleavage of cGAS in human THP-1 cells (Zheng et al., 2018). The further investigation on how NS2b3 or other ZIKV proteins modulate cGAS-STING signaling pathway is needed in future studies.

## ZIKV Antagonizes the JAK-STAT Signaling Pathway

ZIKV infection has been demonstrated to regulate IFN response and its downstream signaling (Grant et al., 2016; Van der Hoek et al., 2017; Wu et al., 2017). JAK1, an important molecule in IFN signaling downstream, was decreased in ZIKV-infected A549 cells. A preliminary study showed that ZIKV NS2b3 protease was responsible for such decrease of JAK1 protein and JAK1 protein was stabilized by proteasome inhibitor MG132, suggesting the proteasome-mediated JAK1 degradation. However, details of relevant mechanisms are needed to uncover how ZIKV NS2b3 modulates JAK1 protein physiology. STAT1 and STAT2 phosphorylation is additionally a critical event in IFN downstream signaling. Strikingly, ZIKV infection prevented the phosphorylation of STAT1 at residue Tyr701 and STAT2 at residue Tyr689 in human A549 and dendritic cells. Specifically, ZIKV NS5 protein has been shown to not only inhibit endogenous STAT1 phosphorylation in HEK293T cells upon IFN-β stimulation but also degrade human STAT2 molecule in multiple cell lines. Further evidence showed that such degradation was mediated by the proteasome because the MG132 can inhibit STAT2 degradation (Grant et al., 2016). However, it is still unknown so far how ZIKV NS5 modulates the JAK-STAT signaling pathway for viral pathogenesis.

## ZIKV Modulates TBK1-Mediated Immune Signaling

There are multiple lines of evidence supporting the modulation of immune molecule TBK1 by ZIKV infection (Wu et al., 2017; Xia et al., 2018; Lin et al., 2019). Endogenous TBK1 phosphorylation and thus IFN response was tremendously inhibited by ZIKV NS1 and NS4B expression in Sendai-infected A549 cells (Wu et al., 2017). ZIKV NS1 and NS4B were shown to associate with TBK1 to reduce TBK1 oligomerization; however, the consequence of such reduction remains unknown. ZIKV NS5 was associated with TBK1 and TRAF6, the latter being a TBK1-binding partner, and thus caused compromised TBK1–TRAF6 association. However, the detailed effect on such NS5-mediated modulation on physiological ZIKV infection remains to be investigated. Additionally, the residue 181 in ZIKV NS1 protein was critical for NS1-TBK1 binding and inhibited RIG-I mediated IFN signaling and production, because the mutation from valine to alanine at amino acid 181 in NS1 abolished TBK1- and IRF3-mediated phosphorylation of RIG-I-2CARD at Ser-172 and Ser-396 respectively, thereby resulting in the inability of RIG-I-mediated IFN response. It was reported that ZIKV NS5 not only decreased phosphorylation IRF3 at Ser-396 by TBK1 in HEK293 cells but also interacted with endogenous IRF3 through MTase domain (Xia et al., 2018), which together inhibited IRF3-mediated IFN response.

## Concluding Remarks

ZIKV became a terrible pathogenic agent since 2015 due to its ability to cause neurogenesis impairment in developing brain and, consequently, microcephaly. Importantly, delayed childhood neurodevelopment and neurosensory alterations in ZIKV-exposed macaques or children have been documented (Nielsen-Saines et al., 2019; Valdes et al., 2019; Raper et al., 2020). Therefore, the existence of short-term and long-term effects of ZIKV infection is calling for further understanding ZIKV biology. Embryonic development, the stage in which ZIKV prefers to invade, is accompanied with complicated neuroimmune regulation involved interactively in mother, placenta, and embryo. The efficient evasion of innate immune defenses by ZIKV is indispensable to facilitate viral infection. Thus, investigation on how ZIKV could evade the host innate immune defenses is an essential topic to understand how ZIKV is able to invade fetal brain. More delicate studies on how ZIKV exerts neuroimmune evasion not only contribute to understanding ZIKV pathogenesis but also offer useful basis for anti-viral drug development.

## Author Contributions

XX wrote the manuscript. JZ contributed to the framework and editing of the manuscript. All authors contributed to the article and approved the submitted version.

## Funding

This work was supported by start-up funding from Kunming Institute of Zoology in Chinese Academy of Sciences to JZ.

## Conflict of Interest

The authors declare that the research was conducted in the absence of any commercial or financial relationships that could be construed as a potential conflict of interest.

## Publisher’s Note

All claims expressed in this article are solely those of the authors and do not necessarily represent those of their affiliated organizations, or those of the publisher, the editors and the reviewers. Any product that may be evaluated in this article, or claim that may be made by its manufacturer, is not guaranteed or endorsed by the publisher.
